# A Real-Time Early Warning System for Monitoring Inpatient Mortality Risk: Prospective Study Using Electronic Medical Record Data

**DOI:** 10.2196/13719

**Published:** 2019-07-05

**Authors:** Chengyin Ye, Oliver Wang, Modi Liu, Le Zheng, Minjie Xia, Shiying Hao, Bo Jin, Hua Jin, Chunqing Zhu, Chao Jung Huang, Peng Gao, Gray Ellrodt, Denny Brennan, Frank Stearns, Karl G Sylvester, Eric Widen, Doff B McElhinney, Xuefeng Ling

**Affiliations:** 1 Department of Health Management, Hangzhou Normal University Hangzhou China; 2 HBI Solutions Inc Palo Alto, CA United States; 3 Department of Cardiothoracic Surgery Stanford University Stanford, CA United States; 4 Clinical and Translational Research Program Betty Irene Moore Children's Heart Center Lucile Packard Children’s Hospital Palo Alto, CA United States; 5 National Taiwan University-Stanford Joint Program Office of AI in Biotechnology Ministry of Science and Technology Joint Research Center for Artificial Intelligence Technology and All Vista Healthcare Taipei Taiwan; 6 Shandong University of Traditional Chinese Medicine Shandong China; 7 Department of Surgery Stanford University Stanford, CA United States; 8 Department of Medicine, Berkshire Medical Center Pittsfield, MA United States; 9 Massachusetts Health Data Consortium Waltham, CA United States

**Keywords:** inpatients, mortality, risk assessment, electronic health records, machine learning

## Abstract

**Background:**

The rapid deterioration observed in the condition of some hospitalized patients can be attributed to either disease progression or imperfect triage and level of care assignment after their admission. An early warning system (EWS) to identify patients at high risk of subsequent intrahospital death can be an effective tool for ensuring patient safety and quality of care and reducing avoidable harm and costs.

**Objective:**

The aim of this study was to prospectively validate a real-time EWS designed to predict patients at high risk of inpatient mortality during their hospital episodes.

**Methods:**

Data were collected from the system-wide electronic medical record (EMR) of two acute Berkshire Health System hospitals, comprising 54,246 inpatient admissions from January 1, 2015, to September 30, 2017, of which 2.30% (1248/54,246) resulted in intrahospital deaths. Multiple machine learning methods (linear and nonlinear) were explored and compared. The tree-based random forest method was selected to develop the predictive application for the intrahospital mortality assessment. After constructing the model, we prospectively validated the algorithms as a real-time inpatient EWS for mortality.

**Results:**

The EWS algorithm scored patients’ daily and long-term risk of inpatient mortality probability after admission and stratified them into distinct risk groups. In the prospective validation, the EWS prospectively attained a c-statistic of 0.884, where 99 encounters were captured in the highest risk group, 69% (68/99) of whom died during the episodes. It accurately predicted the possibility of death for the top 13.3% (34/255) of the patients at least 40.8 hours before death. Important clinical utilization features, together with coded diagnoses, vital signs, and laboratory test results were recognized as impactful predictors in the final EWS.

**Conclusions:**

In this study, we prospectively demonstrated the capability of the newly-designed EWS to monitor and alert clinicians about patients at high risk of in-hospital death in real time, thereby providing opportunities for timely interventions. This real-time EWS is able to assist clinical decision making and enable more actionable and effective individualized care for patients’ better health outcomes in target medical facilities.

## Introduction

### Importance of an Early Warning System

The condition of some hospitalized patients rapidly deteriorates because of either disease progression or imperfect triage and level of care assignment after their admission. Evidence from observational studies show that signs of clinical deterioration (eg, abnormal vital signs) hours before a serious clinical event [1,2] are important predictors. Therefore, we hypothesize that an early warning system (EWS) to identify patients at high risk of subsequent intrahospital death can be an effective tool to improve patient safety and quality of care and also reduce avoidable harm and costs. For patients without a do-not-resuscitate (DNR) order, a warning from such an EWS can activate rapid response teams (RRTs) or medical emergency teams to offer more intensive care and enhanced attention to prevent hospital deaths [3,4]. For those patients with a DNR order, the notification can trigger health caregivers to counsel and work with patients’ families to initiate the end-of-life care and deathbed farewell [5]. Therefore, an EWS to identify and alert caregivers of truly high-risk patients before their deterioration is recognized as an essential step toward the advancement of individualized medical interventions, the improvement of end-of-life patient care quality, and the reduction of unnecessary in-hospital mortality and associated health resource utilization.

### Current Development of an Early Warning System

During the last decade, an increasing number of hospital systems have started to implement EWSs to monitor all adult patients in acute hospital settings and to identify adverse trends and patient deterioration [3,6]. A variety of EWSs have been developed using patients’ postadmission clinical information [2,7-14]. For instance, the widely used VitalPAC Early Warning Score (ViEWS) is calculated from 7 vital sign parameters selected by a thorough literature review and was proven to outperform most other published systems when predicting in-hospital death within 24 hours postobservation [9,10]. However, in addition to using a limited number of parameters empirically selected by experts, we speculate whether such EWSs could be further improved in terms of both sensitivity and specificity by integrating more clinical and nonclinical information (eg, disease diagnoses and social determinant data).

With the rapid growth of hospital adoption of electronic medical record (EMR) systems, other temporal clinical information is becoming available at the point of care and can be used to facilitate the prediction of in-hospital mortality. Several EMR-based risk models have been constructed using surgical record data, laboratory test results, and location transfer information [13-17]. Among these models, clinical utilization factors were rarely considered as potential predictors for inpatient mortality, even though they are valuable features from the aspect of hospital quality measurement.

### Aim of This Study

In this study, we aimed to build and prospectively validate an EMR-based inpatient mortality EWS. We expected that by adopting the machine learning algorithms, this EMR-based EWS could capture previously ignored but useful variables and attain an improved discriminative ability with higher levels of sensitivity and specificity. We evaluated its performance and addressed questions such as how early the system can prospectively alarm for a hospitalized mortality event. We also studied the association between impactful predictors (eg, historical clinical utilizations) and inpatient mortality under various circumstances.

## Methods

### Setting and Patient Population

**Figure 1:**
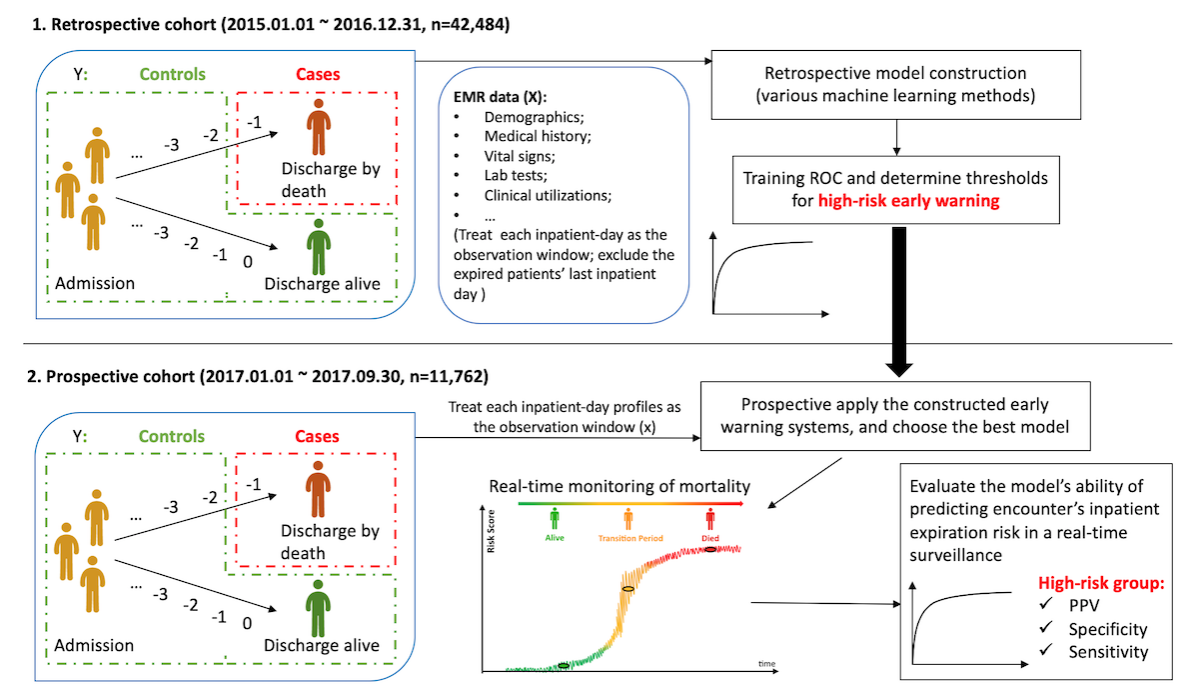
Study design. The early warning system model was built on the retrospective cohort (n=42,484) and validated on the prospective cohort (n=11,762). EMR: electronic medical record; PPV: positive predictive value; ROC: receiver operating characteristic.

The patient population is defined as the patients admitted to two acute hospitals (ie, Berkshire Medical Center and Fairview Hospital) within the Berkshire Health System (BHS), between January 1, 2015 and September 30, 2017. Patients included in the study were those admitted to a medical unit (including the intensive care unit) from either the emergency department (ED) or outpatient clinics, regardless of whether they had DNR orders. The details are shown in the study design workflow (Figure 1). BHS authorized the use of the deidentified data for this research, and thus all personal privacy information was masked during the process of analysis and publication. This study was also exempted from ethics review by the Stanford University institutional review board (September 25, 2018).

### Outcome Variables

Following the rationale that patients who died showed signs of clinical deterioration before death, we identified the cases of our study as the 24-hour period immediately before the day of death for those patients who died and classified all other 24-hour periods as the controls (Figure 1). We used the EMR profile collected before the future 24-hour period as the predictors of the following 24-hour period, making the prediction model capable of estimating the risk of death at least 24 to 48 hours before the event.

### Predictor Variables and Feature Selection

In this study, we defined an inpatient day as a time period between 12:00 am and 11:59 pm in an episode. Within an encounter’s observation window (ie, each inpatient day), candidate predictor variables were extracted from the hospital EMR system, comprising (1) a set of static historical medical variables and (2) a number of dynamic updated postadmission clinical information. By using the medical data cumulatively collected until a certain inpatient day after admission, the risk model was initially designed to predict a patient’s probability of dying in the following inpatient day. Before the machine learning process, we carried out feature selection using both literature review for including impactful feature inclusion and a univariate filtering process for exclusion. As a result, we recruited 680 potential predictors into the subsequent analysis.

### Retrospective Derivation and Prospective Evaluation of the Real-Time Inpatient Mortality Early Warning System

#### Retrospective Model Derivation

At the derivation stage, the real-time inpatient mortality model was constructed on the EMR data collected within a retrospective 2-year period during January 1, 2015 and December 31, 2016, with a total of 42,484 inpatient encounters (Figure 1). At this stage, multiple existing predictive machine learning algorithms (linear and nonlinear) were explored to construct the prediction model, including the tree-based random forest method [18], XGBoost [19], Boosting [20], Support Vector Machine [21], LASSO [22], and K-nearest neighbors [23]. Following this, the predicted outcomes were calibrated to the positive predictive values (PPVs) on the retrospective cohort. This allowed us to calculate the risk score of mortality for each inpatient day during the in-hospital episode and use the quintiles of these calibrated risk scores to stratify risk groups. The propensity score matching was also introduced to investigate the causal relation between high-weight chronic-based risk factors and the inpatient mortality outcome.

#### Prospective Model Evaluation

The constructed models were prospectively evaluated on inpatient admissions for the period January 1, 2017 to September 30, 2017. A total of 11,762 hospitalized patients were assigned an EWS score during this period. The discriminatory power of various algorithms was assessed and compared using the receiver operating characteristic (ROC) curve and the prospectively validated c-statistic. According to the prospective results, the model that attained the best performance was chosen as the proposed EWS. Using the final EWS, we also derived the distribution of inpatient days across the spectrum of the calibrated risk scores and evaluated various risk bins for sensitivity, specificity, and PPVs. On the basis of these determined risk categories (*low*, *intermediate* and *high*), we prospectively explored their subsequent mortality rate using the Kaplan-Meier method and compared their hazard ratios (HRs) using Cox regression. We also conducted subgroup analysis to review the model’s utility on encounters with specific conditions (eg, DNR orders or high clinical costs in the past).

## Results

### Inpatient Mortality Early Warning System Performance on Inpatient-Day Level

**Figure 2:**
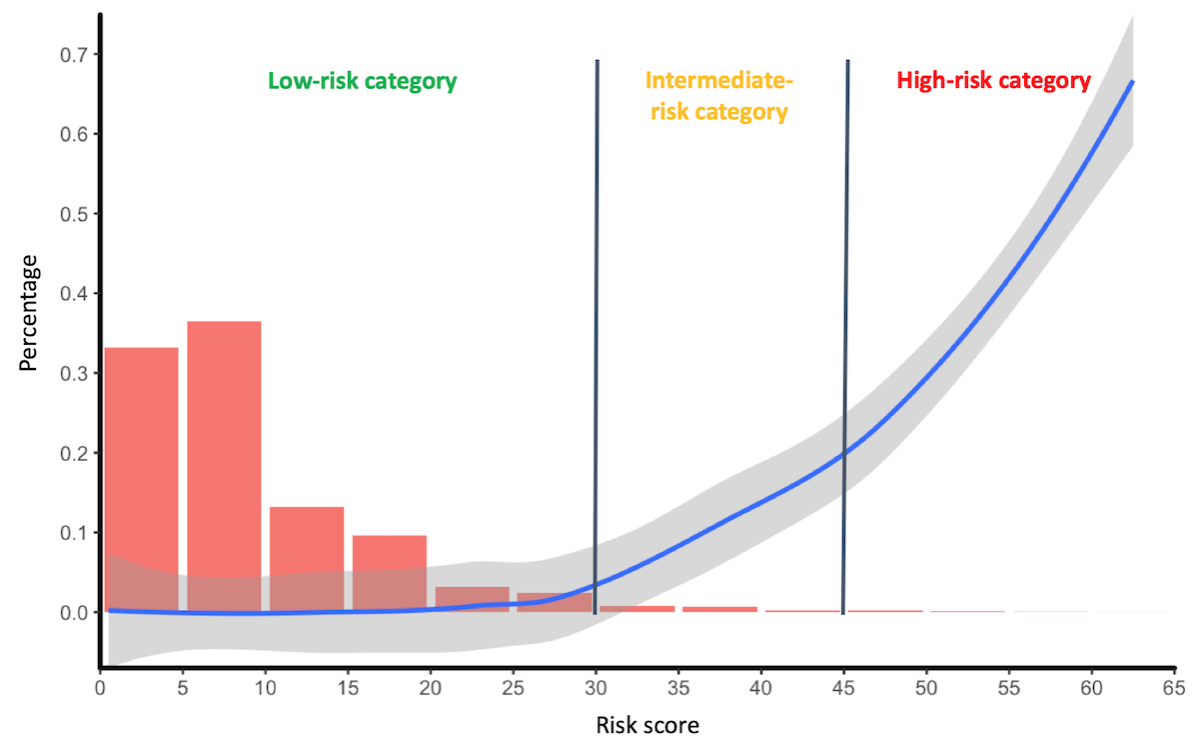
The distribution of inpatient days (the red bar) and positive predictive values (the blue line), coordinated with the inpatient-mortality risk scores on the prospective cohort.

The retrospective and prospective cohorts comprised 42,484 and 11,762 encounters, respectively; 2.34% (993/42,484) and 2.17% (255/11,762) of the patients in these cohorts died during their episode. The demographics and important characteristics of these two cohorts were summarized in Multimedia Appendix 1. After applying the various EWS algorithms to the prospective cohort, we compared their performance as measured by the ROC curve and validated c-statistic. The tree-based random forest algorithm attained the highest predicted c-statistic of 0.884, whereas other machine learning algorithms (linear and nonlinear) attained a predicted c-statistic between 0.511 and 0.867 (Multimedia Appendix 2). Thus, we chose the random forest algorithm–based EWS as the final proposed EWS, where we initially assigned a calibrated risk score to each inpatient day and then stratified these inpatient days into distinct risk groups across the spectrum of risk scores (Figure 2). For a total of 56,588 observed inpatient days, almost 69.66% (39,420/56,588) were located in the *low-risk* percentiles (ie, 0-10), with only 0.09% (35/39,420) of them being cases. Meanwhile, a total of 189 observations fell into the *high-risk* percentiles (ie, ≥45), with 31.2% (59/189) passing away in the subsequent 24 hours (Multimedia Appendix 3).

### Performance of the Early Warning System in Predicting Patients’ Overall Inpatient Mortality

**Figure 3:**
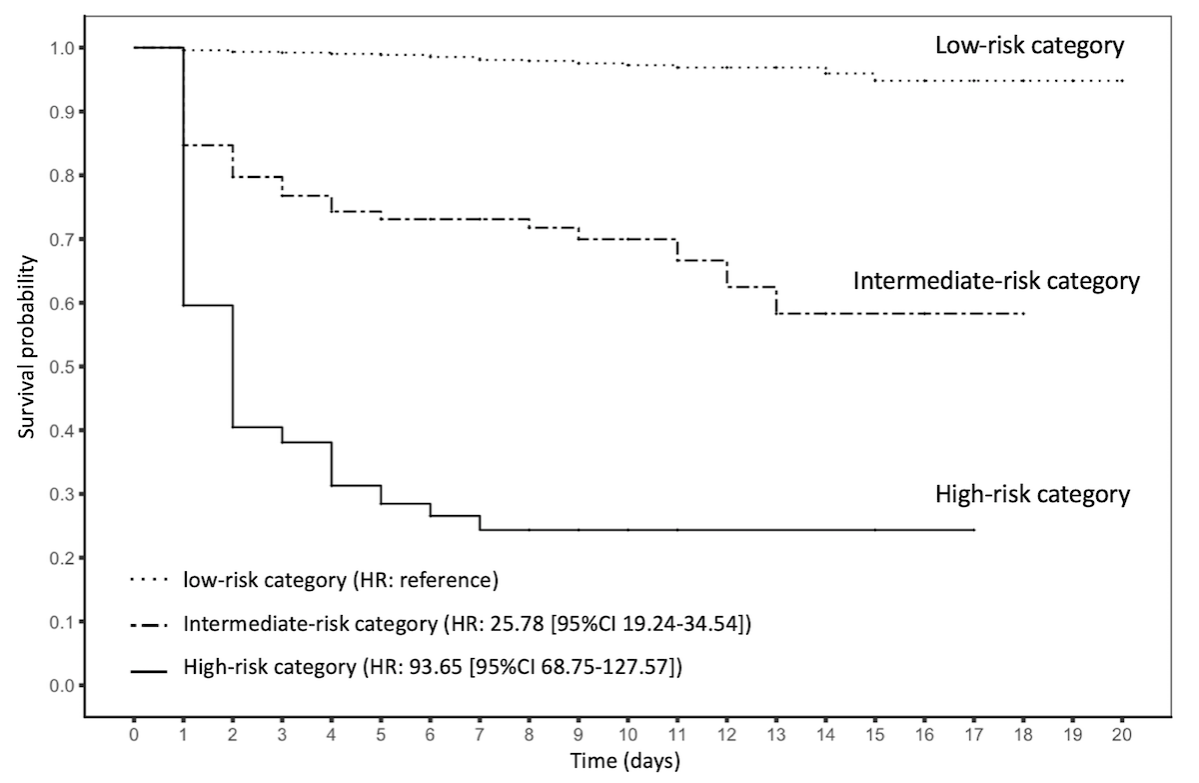
The observed survival curves of the 3 risk categories (encounter-level) stratified by the real-time early warning system in the prospective validation cohort. HR: hazard ratio.

**Figure 4:**
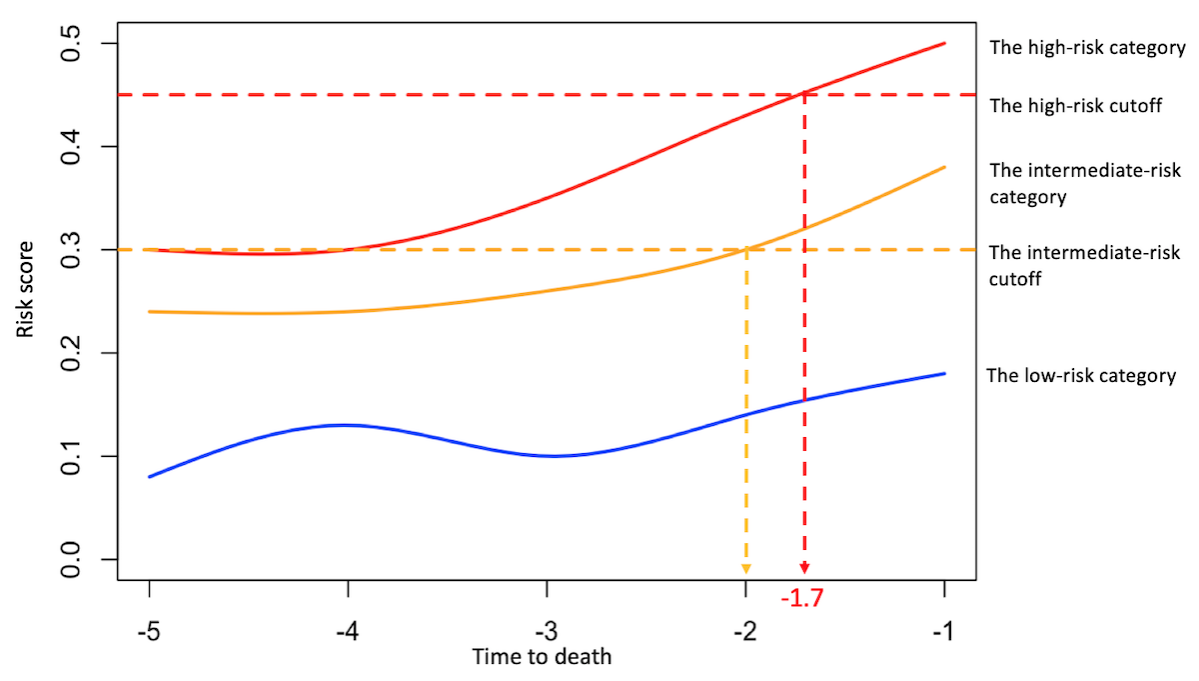
The median real-time risk score curves of the patients who passed away stratified by 3 risk categories of the prospective cohort.

In terms of long-term in-hospital mortality, the proposed EWS model captured 99 encounters with *high risk* of expiration (ie, risk score ≥45) and recognized 327 encounters as *intermediate-risk* individuals (ie, risk score 30-45) at the prospective validation stage (Multimedia Appendix 4). By further tracking the *high-risk* patients’ mortality rate for the subsequent 20 days, we confirmed that the EWS model successfully alerted clinicians to 40% (40/99) of the top risk encounters 24 to 48 hours before their death, notified another 17% (17/99) 48 to 72 hours before their death, and identified the remaining 11% (11/99) 3 to 7 days ahead of their death, making the survival probability drop to 0.24 within 1 week after triggering the alarms (Figure 3). Furthermore, the mortality hazard ratio of the *high-risk* category is as high as 93.65 (95% CI 68.75-127.57) for the subsequent 20-day time period compared with that of the *low-risk* category. In addition, when focusing on the patients who passed away, the results illustrated that the EWS model successfully seized the top 13.3% (34/255) of the population at least 1.7 inpatient days (40.8 hours) before their death (Figure 4). These findings demonstrated that the proposed EWS had powerful discriminative ability to help notify caregivers of inpatient death in the longitudinal scale and assist in clinical decision making.

### Comparison With Currently Used Methods

Several EWSs have already been widely used in current hospital care to provide early warnings of clinical deterioration, such as the ViEWS, the National Early Warning Score, and the Modified Early Warning Score [8,9,24-26]. The shared rationale underlying these common EWSs is that a patient’s deterioration can be estimated with a numeric score derived from a small number (<10) of core signs of physiological function including, but not limited to, heart rate, breathing rate, body temperature, systolic blood pressure, oxygen saturation, urine output, and level of consciousness. Given that the used parameters have been recognized as vital to life (Multimedia Appendix 5), these EWSs can be readily implemented and are expected to have good predictive ability for life-threatening outcomes. However, by using only vital sign abnormalities, the existing methods attain a high specificity with a low sensitivity [27]. Meanwhile, other innovative EWSs have been implemented with better performance by extracting temporal clinical information from EMRs [13-15,17]. In this study, we hypothesize that integration of the real-time EMR datasets with vital signs, laboratory data, disease diagnosis, and clinical utilization indicators shall lead to an EWS with an improved performance in terms of both sensitivity and specificity. Therefore, we compared the proposed EWS with ViEWS, a well-recognized EWS leader, which was proven to outperform most other systems [9,10]. In this comparison, we applied the abbreviated ViEWS tool on the prospective dataset, which achieved a prospective c-statistic of 0.764, a much lower value than that of the EWS model (c-statistic=0.884; Multimedia Appendix 6). Furthermore, when considering only *high-risk* individuals, the EWS achieved a sensitivity of 26.7% (68/255) and a PPV of 69% (68/99), whereas the ViEWS method attained a much lower sensitivity of 13.7% (35/255) and a PPV of only 35% (35/99). When considering both *high* and *intermediate-risk* patients, the EWS attained a sensitivity of 59.2% (151/255) and a PPV of 35.4% (151/426), which were still much higher than that of ViEWS (a sensitivity of 35.7% (91/255) and a PPV of 21.4% (91/426); Multimedia Appendix 6).

### Impactful Predictors in the Developed Early Warning System

We further adopted the Gini impurity [18] as the indicator of the variable importance, as it usually gives a much faster calculation while providing similar results to the out-of-bag permutation measure. By applying the Gini impurity measurement, we recognized 349 impactful predictors for inpatient mortality from the initial 600 input features. We listed the top 50 most significant features in Multimedia Appendix 7.

**Figure 5:**
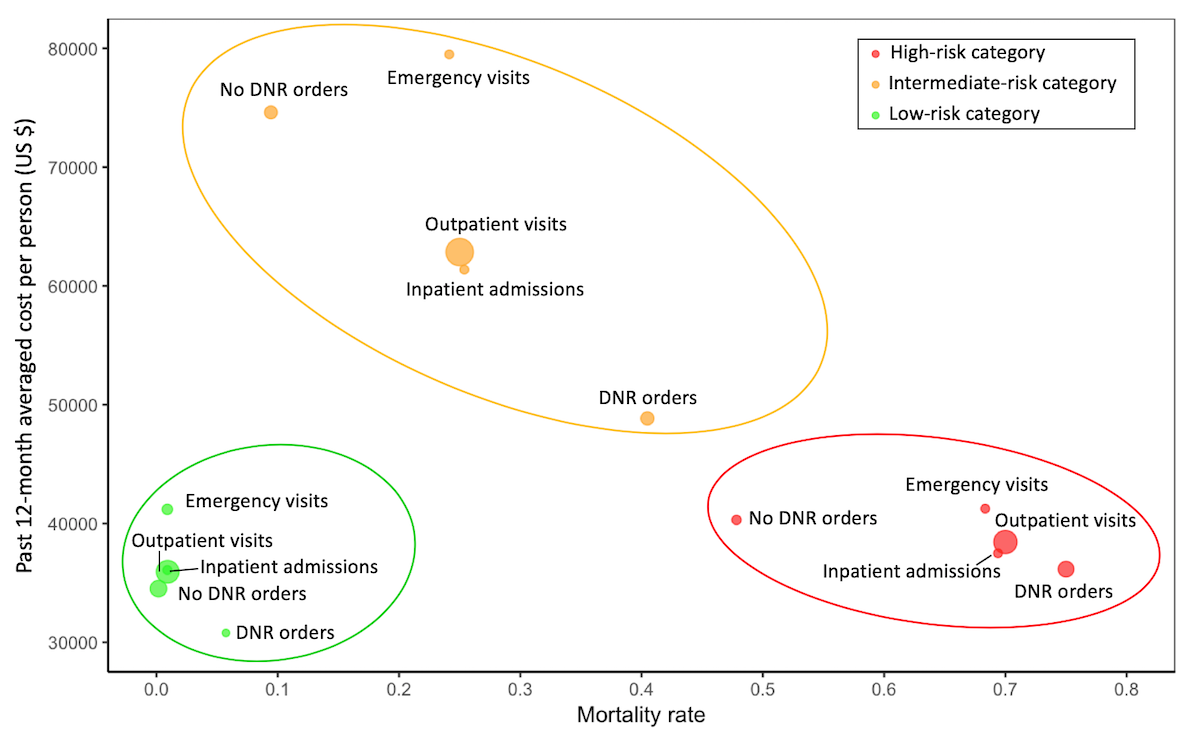
The averaged prior-12-month clinical costs of distinct clinical utilization subgroups, coordinated by their observed mortality rates. Those subgroups are naturally clustered into 3 mortality risk categories of the prospective cohort. Size of each ball: the median of each group. DNR: do-not-resuscitate.

Among these features, the proposed EWS recognized several historical clinical utilization features as highly significant predictors of in-hospital deterioration, including ED visits, inpatient admissions, and outpatient visits and clinical costs in the prior 12 months. We grouped patients by the type (ie, emergency, inpatient and outpatient) of their hospital visits and prospectively compared their averaged prior-12-month clinical costs across the 3 determined risk categories, coordinated by their observed inpatient mortality rate (Figure 5). The results showed that these subgroups aggregated naturally into 3 identified risk clusters when plotting by the dimensions of historical clinical costs and observed mortality rates. For patients estimated as *high risk* of inpatient mortality, subgroups of emergency, inpatient, or outpatient encounters, all had higher observed mortality rates but lower clinical costs than those of the *intermediate-risk* patients. On the contrary, *intermediate-risk* patients had dramatic increase of their prior-12-month clinical costs, especially for patients with emergency visits who ended up with a modest rate of inpatient mortality.

Furthermore, when focusing on these top impactful chronic-based risk factors, we found that only the diagnoses of cardiovascular diseases, congestive heart failure, or renal diseases were still significantly associated with the mortality outcome, whereas other chronic-based features failed to attain significance in terms of odds ratios (ORs) after applying the propensity score matching analysis in our study (Multimedia Appendix 8). The results of the propensity score matching analysis revealed the insignificant independent effects of some targeted chronic risk factors when matched with other significant risk factors. Therefore, we reason that, in the hospital inpatient mortality setting, instead of being the causality of the mortality outcome, some high-weight chronic-based risk factors could be causally related to the acute setting risk factors or interact with other risk factors (such as demographic characteristics), indirectly and interactively contributing to the prediction of the targeted mortality outcome.

### Patients With and Without a Do-Not-Resuscitate Order

We further investigated the EWS model’s discriminative ability in different subgroups of patients with specific diagnoses and conditions (Multimedia Appendix 9), especially patients with and without DNR orders. As confirmed in the validation results, the DNR order patients usually had a much higher inpatient mortality rate than that of the non-DNR order ones (Multimedia Appendix 10). Meanwhile, when looking only at the DNR-order encounters, their mortality rate was still stratified by the 3 distinct risk categories of the EWS; the mortality rate of DNR-order encounters reached its highest value of 75% (57/76) in the *high-risk* category, dropped to 40.5% (68/168) in the *intermediate-risk* category, and plunged to 5.73% (88/1,537) in the *low-risk* category (Multimedia Appendix 10 and Multimedia Appendix 4). This implied that even though some encounters were coded by DNR orders, they still varied significantly in their current in-hospital mortality risk.

## Discussion

### Summary of Principal Findings

In this study, we developed and prospectively validated a real-time EMR-based EWS of inpatient mortality, which predicted encounters’ daily and longitudinal probability of inpatient mortality. With a total of 11,762 hospitalized encounters at the prospective validation stage, this model achieved a c-statistic of 0.884, prognosticated *high risk* of death for 99 encounters during their inpatient stay. For these *high-risk* encounters, 40% (40/99) were confirmed to have passed in the subsequent 24 hours, and 69% (68/99) were confirmed to have passed within 7 days after the notification, resulting in their mortality HR as high as 93.65 (95% CI 68.75-127.57) compared with that of the *low-risk* category. Furthermore, the EWS model successfully prognosticated the death of the top 13.3% (34/255) of the dead patients at least 1.7 days before their death.

In this study, we compared the EWS with the well-recognized EWS tool, ViEWS, and demonstrated that the EWS attained a much higher sensitivity and PPV when giving alerts for the *high-risk* patients. Compared with these existing EWSs, the proposed model involved not only traditional predictors of inpatient mortality, such as vital signs and laboratory data [8,9,28,29], but also valuable historical medical features, such as certain disease diagnoses and clinical utilization indicators, which were usually not included in most previous studies [8,9,14]. However, these inpatient setting features, representing patients’ baseline differences, can contribute indirectly to patients’ distinct hospital mortality rate assessment [30]. In this study, the *intermediate-risk* population in our study, instead of the *high-risk* group, was found to have the highest historical medical costs (Figure 5). This may imply that some of these *high-risk* patients were already coded with DNR orders, directly reducing their clinical costs; others may have deteriorated too rapidly from a healthy status and therefore, never received adequate medical service before death, also resulting in low costs. Therefore, we believe that such historical information in EMR datasets are valuable sources of predictors of inpatient hospital mortality. These risk predictors may interact with other features to facilitate the identification of more true-positive patients, resulting in an improved sensitivity.

### Implications of the Developed Early Warning System

In this study, random forest outperformed other commonly used algorithms on the prospective cohort. As an ensemble tree-based method, random forest has been proven to have high accuracy as it overcomes overfitting by selecting random subsets of features to build smaller trees and is able to handle potential errors caused by unbalanced case-control datasets (in this case, inpatient mortality, where only a relatively small proportion of patients suffered in-hospital death) [31]. In addition, random forest makes no assumptions regarding the predictor features’ distributions and correlations and is able to capture features with weak effects as well as their high-level interactions, thus making it suitable for our EMR-based prediction based on multiple correlated covariates [32]. Along with the massively increased data, another well-recognized method, deep learning, is popularly used because of the recent breakthroughs in algorithm development. However, deep learning does not necessarily perform better than linear and nonlinear machine learning methods, as it usually returns a result that is difficult to interpret for domain specialists, and it is more computationally consuming and expensive, especially in the model development stage [33].

It is worth noting that in the prospective cohort, 31 of the 99 patients who were given alerts for *high risk* of inpatient mortality survived through the entire hospital encounter. After investigating those recovered patients, we found that most of them (25/31) received diagnoses of either cardiovascular diseases, renal disease, cancer, lung disease, or acute cerebrovascular disease, which implied severe acute or chronic disease conditions as well as the requirement of more intense care during their hospital stay. In such an early warning context, it is demonstrated elsewhere that sensitivity and PPV are always considered important indicators; however, these patients who were alerted as high risk yet later recovered may not necessarily be treated as falsely alarmed individuals, as caregivers could always provide clinical intervention or treatment to these high-risk patients during their deterioration process and potentially prevent their death events from occurring [34]. Thus, from the perspective of inpatient mortality reduction and clinical care promotion, it would be valuable to track and summarize those efficient interventions or treatments provided to these high-risk but recovered patients, facilitating evidence-based clinical decision making and individualized care planning for other high-risk patients.

Many of the diseases currently being treated in the wards are major injuries, and these patients could become potential confounders when predicting the hospital inpatient mortality. However, patients with such major injuries are often difficult to define in the EMR system, as there is an issue with the preciseness of their diagnoses when using the International Classification of Diseases (ICD) codes. In this study, taking fracture as an example, we found that when using the standard ICD-10 definitions (Multimedia Appendix 11), the proportion of patients with a fracture diagnosis was relatively small in both the overall cohort and the *high-risk* category (ie, overall: 5.42% [639/11,762]; *high-risk* category: 8.10% [8/99]) and the OR was also not significantly different between cases and controls (OR 1.17; 95% CI 0.67-1.89). To address and verify the impact of major injuries as confounding factors, we hypothesized that young patients are more likely to die of major injuries, whereas the older patients mainly suffered from other severe conditions, and thus, we used age as a summarizing indicator of major injuries in our dataset. After investigating the age-stratified mortality across the identified risk categories (*high*, *intermediate*, and *low*), we revealed that instead of young patients, most true positives in the identified *high* and *intermediate* *-risk* categories were older than 60 years (Multimedia Appendix 11), who were less likely to die of accidents or major injuries. Therefore, we concluded that in our study cohort, major injuries did not have a significant impact on the inpatient mortality prediction, but we should be careful to consider this confounding effect for applications in the future.

### Utilization and Benefits of the Early Warning System

**Figure 6:**
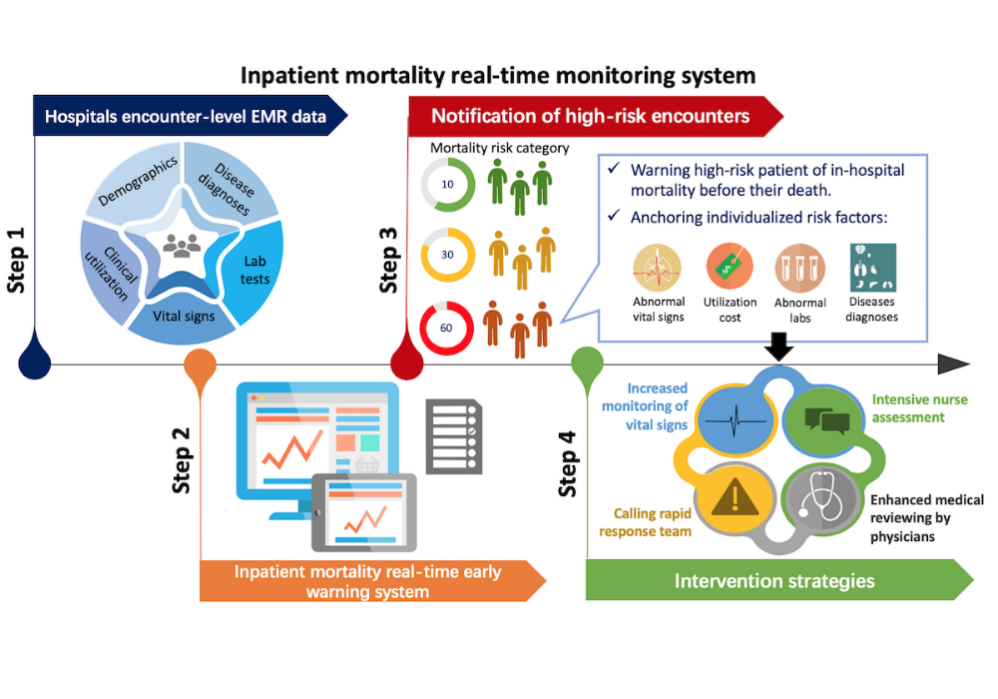
The implementation framework and workflow of the real-time early warning system (EWS), demonstrated in 4 steps: (1) import patient encounters’ electronic medical record (EMR) data into the EWS, (2) monitor their inpatient mortality risk scores every 15 min in the user interface after the deployment, (3) use predetermined thresholds to predict the encounters with high risk and intermediate risk of inpatient mortality in a real-time scenario, and (4) highlight or pop up individualized impactful risk factors to help design and implement the subsequent individualized intervention.

Previous studies have developed specific in-hospital mortality models suitable for a certain disease or condition, such as acute myocardial infarction [35,36] and congestive heart failure [37]. Compared with these models, the EWS model can be universally applied to all hospitalized patients without restricting them to a certain disease diagnosis. To assist clinical decision making, it can automatically send notifications to physicians and RRT when patients exceed the high-risk threshold, offering a chance at earlier detection of acute events. Furthermore, we can provide clinicians with the real-time risks and specific alerts of the impactful risk factors that the deteriorating patient has and give clinicians suggestions of individualized follow-up health care plans, such as increased monitoring of vital signs, intensive nurse assessments of the patient’s condition, and enhanced medical review by physicians [38] (Figure 6).

Compared with most studies focusing only on patients without DNR orders, the EWS targeted all hospitalized patients regardless of their status of DNR orders. In our study, we found that patients with DNR orders can still be differentiated in terms of their inpatient mortality risk (Multimedia Appendix 10). Previous studies also observed that hospitals’ DNR rates could influence the inpatient mortality outcomes in different ways [39]. Therefore, DNR orders do not necessarily indicate the imminent death of the patients in hospital, and the early warning of their death event is important to help the palliative care providers offer supportive services for both patients and their families, such as relieving patients from the symptoms and stress of the illness and letting the family prepare for the deathbed farewell and bereavement. On the other hand, when considering non–DNR-order patients, the identification of their high-risk status could trigger an early warning, activate in-hospital RRTs to a more intensive intervention, and provide a chance to reduce the death or cardiac arrest rate.

In previous studies, limited evidence has been provided to support the conclusion that EWSs have a straightforward effect on the reduction of mortality and cardiac arrests [27,40,41]. With the deployment of the EWS in the BHS hospitals, we will investigate the EWS’s long-term benefit on patient health and resource utilization outcomes.

### Limitations

The proposed EWS is built on the EMR data from hospitals located in a relatively small region, and thus the model may not be directly applied to other regions and clinical settings. However, we established the framework and detailed workflow for the construction and validation of the EMR-based inpatient mortality EWS, which can easily be migrated to much broader settings and bigger datasets. In addition, we also consider patient-level social determinants as important and potential data source for in-hospital mortality prediction as most of them are long-term prognosis factors influencing the mortality outcome. Therefore, incorporating such data in the future will make the next-generation EWS model more compelling and robust.

### Conclusions

In this study, by using modern machine learning algorithms, we have developed and prospectively validated an EWS for forecasting inpatient mortality based on patients’ EMR data. This EWS prospectively achieved a high predictive accuracy in the validation stage. As a real-time surveillance system that will be integrated into the target medical facilities to assist clinical decision making in the near future, the EWS could trigger an early notification for the patients at high risk of in-hospital mortality, thereby letting clinicians initiate intensive care before the acute event and provide a chance of individualized management to improve the quality of health care.

## Appendix

Multimedia Appendix 1 Summarized demographics and baseline characteristics.

Multimedia Appendix 2 The receiver operating characteristic curves of various algorithms at the prospective validation stage.

Multimedia Appendix 3 The performance of the inpatient mortality early warning system on the prospective cohort, summarized in inpatient-day level positive predictive value, sensitivity, specificity, and relative risk.

Multimedia Appendix 4 The patient distribution in the 3 risk categories identified by the early warning system in the prospective validation cohort.

Multimedia Appendix 5 Summary of variables used in various currently used early warning systems.

Multimedia Appendix 6 The performance comparison between the early warning system model and the well-recognized VitalPAC Early Warning Score method on the prospective dataset.

Multimedia Appendix 7 The top 50 most important features contributed to the inpatient mortality early warning system.

Multimedia Appendix 8 Odds ratios of the impactful chronic-based predictors before and after the propensity score matching analysis.

Multimedia Appendix 9 The observed mortality rates in distinct patient subgroups, stratified by the low-risk (blue), intermediate-risk (yellow), and high-risk (red) categories identified by the early warning system on the prospective cohort.

Multimedia Appendix 10 The inpatient mortality rate of do-not-resuscitate (DNR)-order (orange) and non–DNR-order (blue) populations in the 3 risk categories of the prospective cohort.

Multimedia Appendix 11 Age-stratified mortality across the identified risk categories.

## Abbreviations

BHS: Berkshire Health System
DNR: do-not-resuscitate
ED: emergency department
EMR: electronic medical record
EWS: early warning system
HR: hazard ratio
ICD: International Classification of Diseases
OR: odds ratio
PPV: positive predictive value
ROC: receiver operating characteristic
RRT: rapid response team
ViEWS: VitalPAC Early Warning Score
