# Supplementary material for: A Real-Time Early Warning System for Monitoring Inpatient Mortality Risk: Prospective Study Using Electronic Medical Record Data
Source: J Med Internet Res. 2019 Jul 5;21(7):e13719. doi: 10.2196/13719 (PMC6640073; doi:10.2196/13719)
Supplement: Multimedia Appendix 1 [file jmir_v21i7e13719_app1.docx]

Appendix 1. Summarized demographics and baseline characteristics.

|  | **Retrospective cohort** | **Prospective cohort** |
| --- | --- | --- |
|  | (N=42,484) | (N=11,762) |
| Encounters discharged by death | 993 (2.34%) | 255 (2.17%) |
| **Demographics** |  |  |
| Age | 59.90 (21.65) | 60.56 (21.40) |
| Gender (female) | 21,509 (50.63%) | 5835 (49.61%) |
| **Historical medical variables** |  |  |
| Patient's estimated cost last year | 28706.84 (44454.38) | 34797.01 (46871.7) |
| Emergency visits last year | 1 (0-4) | 2 (0-4) |
| Inpatient admissions last year | 1 (0-3) | 2 (1-4) |
| Inpatient length of days last year | 4 (3-6) | 4 (3-6) |
| Outpatient visits last year | 11 (2-29) | 15 (5-34) |
| Disorders of lipid metabolism | 4416 (10.39%) | 1585 (13.48%) |
| Anemia | 6012 (14.15%) | 2016 (17.14%) |
| Cardiovascular diseases | 19443 (45.77%) | 6251 (53.15%) |
| Renal failure | 4259 (10.02%) | 1355 (11.52%) |
| Type 2 diabetes | 7449 (17.53%) | 2399 (20.4%) |
| **Vital Signs** |  |  |
| SpO2 (%) | 95.93 (2.69) | 95.93 (2.35) |
| Temperature (°F) | 98.18 (0.65) | 98.21 (0.57) |
| Respiration per minute | 17.43 (2.19) | 17.39 (1.99) |
| Pulse per minutes | 80.94 (13.05) | 80.99 (11.39) |
| Blood Pressure Systolic | 129.25 (26.97) | 128.55 (27.38) |
| Blood Pressure Diastolic | 71.34 (17.88) | 71.70 (24.57) |
| **Laboratory Tests** |  |  |
| Sodium (mEq/L) | 139.56 (3.19) | 139.39 (3.22) |
| Glucose (mg/dL) | 122.48 (45.18) | 121.47 (44.29) |
| Hematocrit (g/dL) | 37.71 (5.58) | 37.94 (5.76) |
| Potassium (mEq/L) | 4.07 (0.41) | 4.05 (0.41) |
| Red Blood Cell Count (m/ul) | 4.12 (0.64) | 4.15 (0.67) |
| White Blood Cell Count (10^3^ cells/mm^3^) | 9.10 (4.38) | 8.96 (4.7) |
| Blood Urea Nitrogen (mg/dL) | 20.50 (13.89) | 19.76 (13.35) |
| Chloride (mEq/L) | 104.15 (4.31) | 103.60 (4.37) |
| Creatinine (mg/dL) | 1.09 (0.83) | 1.07 (0.83) |
| Hemoglobin (gm/dl) | 12.32 (2) | 12.41 (2.08) |
| Anion Gap | 8.62 (2.27) | 9.14 (2.22) |
| Platelets (10^3^ cells/mm^3^) | 242.70 (85.96) | 240.14 (86.23) |
| Estimated Glomerular Filtration Rate (mL/min/1.73 m^2^) | 54.36 (10.39) | 54.66 (10.31) |

Data presented as number (% of column total), mean (standard deviation), or median (1st quartile, 3rd quartile] unless otherwise specified.
