# Supplementary material for: A Real-Time Early Warning System for Monitoring Inpatient Mortality Risk: Prospective Study Using Electronic Medical Record Data
Source: J Med Internet Res. 2019 Jul 5;21(7):e13719. doi: 10.2196/13719 (PMC6640073; doi:10.2196/13719)

Appendix 2: The receiver operating characteristic curves of various algorithms at the prospective validation stage.


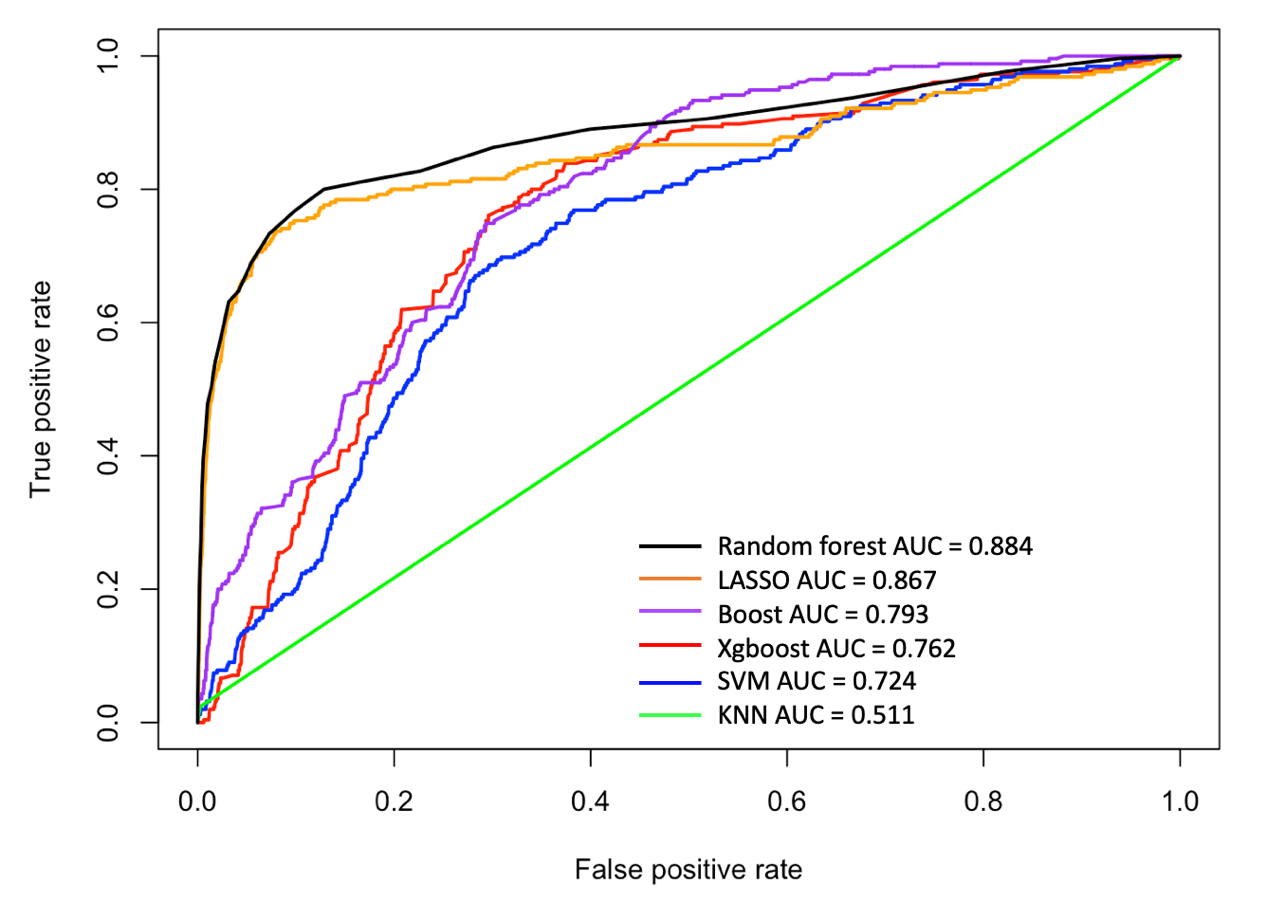

Supplement: Multimedia Appendix 2 [file jmir_v21i7e13719_app2.docx]
