# Supplementary material for: A Real-Time Early Warning System for Monitoring Inpatient Mortality Risk: Prospective Study Using Electronic Medical Record Data
Source: J Med Internet Res. 2019 Jul 5;21(7):e13719. doi: 10.2196/13719 (PMC6640073; doi:10.2196/13719)
Supplement: Multimedia Appendix 3 [file jmir_v21i7e13719_app3.docx]

Appendix 3: The performance of the inpatient mortality early warning system on the prospective cohort, summarized in inpatient-day level positive predictive value, sensitivity, specificity, and relative risk.

|  | 0~10 | 10~15 | 15~20 | 20~25 | 25~30 | 30~35 | 35~40 | 40~45 | 45~50 | 50~55 | 55~60 | 60~65 | Total |
| --- | --- | --- | --- | --- | --- | --- | --- | --- | --- | --- | --- | --- | --- |
| Number of Observations | 39,420 | 7450 | 5424 | 1793 | 1355 | 460 | 351 | 146 | 103 | 57 | 22 | 7 | 56,588 |
| Cases | 35 | 13 | 20 | 22 | 27 | 16 | 33 | 30 | 29 | 15 | 10 | 5 | 255 |
| PPV | 0.09% | 0.17% | 0.37% | 1.23% | 1.99% | 3.48% | 9.40% | 20.55% | 28.16% | 26.32% | 45.45% | 71.43% | 0.45% |
| Observation% | 69.66% | 13.17% | 9.59% | 3.17% | 2.39% | 0.81% | 0.62% | 0.26% | 0.18% | 0.10% | 0.04% | 0.01% | 100.00% |
| Sensitivity | 13.73% | 5.10% | 7.84% | 8.63% | 10.59% | 6.27% | 12.94% | 11.76% | 11.37% | 5.88% | 3.92% | 1.96% | 100.00% |
| Specificity | 69.91% | 13.20% | 9.59% | 3.14% | 2.36% | 0.79% | 0.56% | 0.21% | 0.13% | 0.07% | 0.02% | 0.00% | 100.00% |
| Relative Risk | 0.20 | 0.39 | 0.82 | 2.72 | 4.42 | 7.72 | 20.86 | 45.60 | 62.48 | 58.40 | 100.87 | 158.51 | 1 |
