# Supplementary material for: A Real-Time Early Warning System for Monitoring Inpatient Mortality Risk: Prospective Study Using Electronic Medical Record Data
Source: J Med Internet Res. 2019 Jul 5;21(7):e13719. doi: 10.2196/13719 (PMC6640073; doi:10.2196/13719)
Supplement: Multimedia Appendix 4 [file jmir_v21i7e13719_app4.docx]

Appendix 4: The patient distribution in the 3 risk categories identified by the early warning system in the prospective validation cohort.

| Category | Low-risk | | | Intermediate-risk | | | High-risk | | |
| --- | --- | --- | --- | --- | --- | --- | --- | --- | --- |
| DNR order | Yes | No | Total | Yes | No | Total | Yes | No | Total |
| Expired | 88 | 16 | 104 | 68 | 15 | 83 | 57 | 11 | 68 |
| Not expired | 1449 | 9783 | 11,232 | 100 | 144 | 244 | 19 | 12 | 31 |
| Total | 1537 | 9799 | 11,336 | 168 | 159 | 327 | 76 | 23 | 99 |
