# Supplementary material for: A Real-Time Early Warning System for Monitoring Inpatient Mortality Risk: Prospective Study Using Electronic Medical Record Data
Source: J Med Internet Res. 2019 Jul 5;21(7):e13719. doi: 10.2196/13719 (PMC6640073; doi:10.2196/13719)
Supplement: Multimedia Appendix 5 [file jmir_v21i7e13719_app5.docx]

Appendix 5: Summary of variables used in various currently used early warning systems.

| **Early warning systems** | **Variables** | **Reference** |
| --- | --- | --- |
| **VitalPAC Early Warning Score (ViEWS)** | **Vital sign:** | [1] |
|  | *Pulse (bpm)* |  |
|  | *Breathing rate (bpm)* |  |
|  | *Temperature (◦C)* |  |
|  | *Systolic BP (mmHg)* |  |
|  | *SaO_2_ (%) (aterial oxygen saturation)* |  |
|  | *Inspired O_2_* |  |
|  | *Level of consciousness CNS (use AVPU scale, alert, voice, pain, unresponsive)* |  |
| **NEWS (National Early Warning Score)** | **Vital sign:** | [2] |
|  | *Respiration rate (bpm)* |  |
|  | *SpO_2_ (%)* |  |
|  | *Any supplemental oxygen?* |  |
|  | *Temperature (°C)* |  |
|  | *Systolic BP (mmHg)* |  |
|  | *Heart/pulse rate (bpm)* |  |
|  | *Level of consciousness (AVPU system)* |  |
| **MEWS (Modified Early Warning Score)** | **Vital sign:** | [3] |
|  | *Systolic BP (mmHg)* |  |
|  | *Heart rate (bpm)* |  |
|  | *Respiration rate (breaths per min)* |  |
|  | *Temperature (°C)* |  |
|  | *AVPU score* |  |
| **SEWS chart** | **Vital sign** | [4] |
|  | *Heart rate (bpm)* |  |
|  | *Respiration rate (bpm)* |  |
|  | *Systolic BP (mmHg)* |  |
|  | *AVPU score* |  |
|  | *Temperature (°C)* |  |
|  | *SaO_2_* |  |
| **HOTEL score** | **Vital sign** | [5] |
|  | *Hypotension* |  |
|  | *Oxygen saturation* |  |
|  | *Temperature* |  |
|  | *ECG abnormality* |  |
|  | *Loss of independence* |  |
| **A Prediction Model for Adverse Outcomes on the Wards** | **Vital sign, demographic, and laboratory data** | [6] |
|  | *Time (hours)* |  |
|  | *Prior ICU stay (1=Yes, 0=No)* |  |
|  | *Heart rate (beats/min)* |  |
|  | *Diastolic blood pressure (mm Hg)* |  |
|  | *Respiratory rate (breaths/min)* |  |
|  | *Oxygen saturation (%)* |  |
|  | *Temperature (°C)* |  |
|  | *Mental status (AVPU)* |  |
|  | *On room air (1=Yes, 0=No)* |  |
|  | *Age (years)* |  |
|  | *BUN (mg/dL)* |  |
|  | *Anion gap (mEq/L)* |  |
|  | *Hemoglobin (g/dL)* |  |
|  | *Platelet count (K/uL)* |  |
|  | *Potassium (mEq/L)* |  |
|  | *White blood cell count (K/uL)* |  |
| **Acute Laboratory Risk of Mortality Score (ALaRMS)** | **Age** | [7] |
|  | **Gender** |  |
|  | **23 Laboratory variables** |  |
|  | *Albumin (g/dL)* |  |
|  | *AST (U/L)* |  |
|  | *Total bilirubin (mg/dL)* |  |
|  | *Calcium (mg/dL)* |  |
|  | *Creatinine (mg/dL)* |  |
|  | *pro-BNP (pg/dL)* |  |
|  | *BNP* |  |
|  | *Glucose (mg/dL)* |  |
|  | *K (mEq/L)* |  |
|  | *Na (mEq/L)* |  |
|  | *Alkaline phos (U/L)* |  |
|  | *BUN (mg/dL)* |  |
|  | *pH Arterial* |  |
|  | *PO_2_ Arterial (mm Hg)* |  |
|  | *pCO_2_ Arterial (mm Hg)* |  |
|  | *PTT* |  |
|  | *PT INR* |  |
|  | *Bands (%)* |  |
|  | *Hemoglobin (g/dL)* |  |
|  | *Platelets (10^9^/L)* |  |
|  | *WBC (1000/mm^3^ )* |  |
|  | *Troponin I (ng/mL) or CPK MB (ng/mL)* |  |
| **Real-time prediction of mortality** | **Administrative data** | [8] |
|  | **Laboratory data** |  |
| **A continuous measure of patient condition using EHR** | **26 clinical measurements from four categories** | [9] |
|  | **nursing assessments** |  |
|  | **vital signs** |  |
|  | **laboratory results** |  |
|  | **cardiac rhythms** |  |

**Reference**

1. Prytherch DR, Smith GB, Schmidt PE, Featherstone PI. ViEWS-Towards a national early warning score for detecting adult inpatient deterioration. Resuscitation. 2010;81:932–7.

2. Smith GB, Prytherch DR, Meredith P, Schmidt PE, Featherstone PI. The ability of the National Early Warning Score (NEWS) to discriminate patients at risk of early cardiac arrest, unanticipated intensive care unit admission, and death. Resuscitation. 2013;84:465–70.

3. Subbe CP, Kruger M, Rutherford P, Gemmel L. Validation of a modified Early Warning Score in medical admissions. QJM. 2001;94:521–6.

4. Paterson R, MacLeod DC, Thetford D, Beattie A, Graham C, Lam S, et al. Prediction of in-hospital mortality and length of stay using an early warning scoring system: clinical audit. Clin Med. 2006;6:281–4.

5. Wheeler I, Price C, Sitch A, Banda P, Kellett J, Nyirenda M, et al. Early warning scores generated in developed healthcare settings are not sufficient at predicting early mortality in Blantyre, Malawi: a prospective cohort study. PLoS One. 2013;8:e59830.

6. Churpek MM, Yuen TC, Park SY, Gibbons R, Edelson DP. Using Electronic Health Record Data to Develop and Validate a Prediction Model for Adverse Outcomes in the Wards. Crit Care Med. 2014;42:841–8. doi:10.1097/CCM.0000000000000038.

7. Tabak YP, Sun X, Nunez CM, Johannes RS. Using electronic health record data to develop inpatient mortality predictive model: Acute Laboratory Risk of Mortality Score (ALaRMS). J Am Med Informatics Assoc. 2014;21:455–63. doi:10.1136/amiajnl-2013-001790.

8. Cai X, Perez-Concha O, Coiera E, Martin-Sanchez F, Day R, Roffe D, et al. Real-time prediction of mortality, readmission, and length of stay using electronic health record data. J Am Med Inform Assoc. 2015; December 2014:ocv110. doi:10.1093/jamia/ocv110.

9. Rothman MJ, Rothman SI, Beals J. Development and validation of a continuous measure of patient condition using the Electronic Medical Record. J Biomed Inform. 2013;46:837–48.
