# Supplementary material for: A Real-Time Early Warning System for Monitoring Inpatient Mortality Risk: Prospective Study Using Electronic Medical Record Data
Source: J Med Internet Res. 2019 Jul 5;21(7):e13719. doi: 10.2196/13719 (PMC6640073; doi:10.2196/13719)

Appendix 6: The performance comparison between the early warning system model and the well-recognized VitalPAC Early Warning Score

method on the prospective dataset.

| Methods | Predictors | c-statistics in our dataset | | forecast high-risk encounters | | | forecast high- and intermediate-risk encounters | | |
| --- | --- | --- | --- | --- | --- | --- | --- | --- | --- |
|  |  | Inpatient day level | Real time | PPV | Specificity | Sensitivity | PPV | Specificity | Sensitivity |
| The EWS model | EMR-based predictors | 0.884 | 0.878 | 68.69% | 99.73% | 26.67% | 35.45% | 97.61% | 59.22% |
| ViEWS | Six Vital signs and level of consciousness | 0.764 | 0.76 | 35.35% | 99.44% | 13.73% | 21.36% | 97.09% | 35.69% |


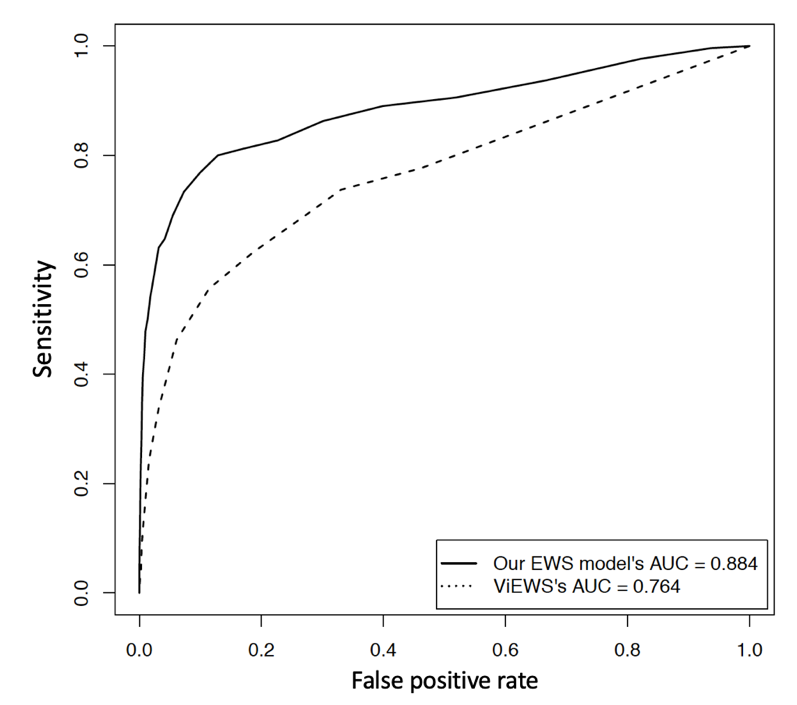

Supplement: Multimedia Appendix 6 [file jmir_v21i7e13719_app6.docx]
