# Supplementary material for: A Real-Time Early Warning System for Monitoring Inpatient Mortality Risk: Prospective Study Using Electronic Medical Record Data
Source: J Med Internet Res. 2019 Jul 5;21(7):e13719. doi: 10.2196/13719 (PMC6640073; doi:10.2196/13719)
Supplement: Multimedia Appendix 7 [file jmir_v21i7e13719_app7.docx]

Appendix 7: The top 50 most important features contributed to the inpatient mortality early warning system.

|  | **Odds Ratio** | **CI1** | **CI2** | **P Value** |
| --- | --- | --- | --- | --- |
| **Demographics** |  |  |  |  |
| Age |  |  |  |  |
| 18-34 | 0.05 | 0.02 | 0.09 | <.001 |
| 35-49 | 0.18 | 0.11 | 0.26 | <.001 |
| 50-64 | 0.53 | 0.43 | 0.64 | <.001 |
| 65-74 | 1.36 | 1.15 | 1.61 | <.001 |
| 75-84 | 2.16 | 1.85 | 2.52 | <.001 |
| >85 | 3.10 | 2.67 | 3.58 | <.001 |
| **Chronic Disease Diagnosis** |  |  |  |  |
| Cardiovascular diseases | 2.74 | 2.07 | 3.68 | <.001 |
| Congestive heart failure | 2.48 | 1.85 | 3.29 | <.001 |
| Myocardial infarction | 1.96 | 1.51 | 2.53 | <.001 |
| Cancer | 2.56 | 1.66 | 3.78 | <.001 |
| Renal disease | 2.16 | 1.58 | 2.90 | <.001 |
| Lung disease | 1.46 | 1.09 | 1.93 | 0.01 |
| Acute cerebrovascular disease | 1.52 | 1.05 | 2.13 | 0.02 |
| Complications of surgical procedures or medical care | 1.66 | 1.16 | 2.31 | 0.004 |
| **Utilization** |  |  |  |  |
| Total inpatient days in last 12 months | 2.22 | -0.38 | 4.83 | 0.09 |
| Emergency visits in last 12 months | -2.24 | -3.23 | -1.26 | <.001 |
| Inpatient admissions in last 12 months | -0.55 | -1.12 | 0.01 | 0.06 |
| Outpatient visits in last 12 months | 3.83 | 0.44 | 7.23 | 0.03 |
| Cost in last 12 months | 3041.26 | -2775.05 | 8857.56 | 0.31 |
| **Vital Sign** |  |  |  |  |
| Pulse per minutes in last 24 hours |  |  |  |  |
| Low | 0.80 | 0.32 | 1.65 | 0.60 |
| High | 3.98 | 3.42 | 4.64 | <.001 |
| Respiration per minute in last 24 hours |  |  |  |  |
| Low | 2.37 | 1.97 | 2.83 | <.001 |
| High | 28.87 | 24.77 | 33.64 | <.001 |
| Temperature (°F) |  |  |  |  |
| <98 | 1.16 | 1.01 | 1.34 | 0.04 |
| >98.6 | 3.97 | 3.36 | 4.68 | <.001 |
| SpO2 |  |  |  |  |
| <95 | 2.63 | 2.29 | 3.03 | <.001 |
| Blood Pressure Diastolic in last 24 hours |  |  |  |  |
| Low | 1.92 | 1.54 | 2.41 | <.001 |
| High | 0.71 | 0.49 | 0.98 | 0.05 |
| Blood Pressure Systolic in last 24 hours |  |  |  |  |
| Low | 2.30 | 1.94 | 2.74 | <.001 |
| High | 0.57 | 0.45 | 0.70 | <.001 |
| **Laboratory Tests** |  |  |  |  |
| Potassium (mEq/L) |  |  |  |  |
| >5 | 3.16 | 2.34 | 4.22 | <.001 |
| <3.5 | 1.87 | 1.42 | 2.45 | <.001 |
| AST (U/L) |  |  |  |  |
| >35 | 1.68 | 1.25 | 2.25 | <.001 |
| Mean Platelet Volume (fL) |  |  |  |  |
| >11.8 | 1.57 | 1.15 | 2.11 | <.001 |
| <7.2 | 0.70 | 0.52 | 0.93 | 0.02 |
| Hematocrit (%) |  |  |  |  |
| >52 (men); >47 (women) | 1.32 | 0.71 | 2.24 | 0.34 |
| <40 (men); <36 (women) | 4.22 | 3.09 | 5.86 | <.001 |
| Creatinine (mg/dL) |  |  |  |  |
| >1.3 | 4.55 | 3.51 | 5.90 | <.001 |
| <0.7 | 2.63 | 1.60 | 4.09 | <.001 |
| Total Bilirubin (mg/dL) |  |  |  |  |
| >1.2 | 2.30 | 1.58 | 3.27 | <.001 |
| <0.3 | 0.64 | 0.04 | 2.86 | 0.66 |
| Sodium (mEq/L) |  |  |  |  |
| >145 | 5.83 | 4.39 | 7.69 | <.001 |
| <136 | 1.80 | 1.35 | 2.38 | <.001 |
| Troponin I (ng/ml) |  |  |  |  |
| >0.1 | 5.30 | 4.06 | 6.92 | <.001 |
| Plasma Glucose (mg/dL) |  |  |  |  |
| >105 | 3.01 | 2.15 | 4.33 | <.001 |
| <70 | 2.17 | 1.51 | 3.03 | <.001 |
| CO2 (mEq/L) |  |  |  |  |
| >28 | 1.98 | 1.39 | 2.74 | <.001 |
| <23 | 4.56 | 3.42 | 6.02 | <.001 |
| Mean Corpuscular Hemoglobin Concentration (MCHC) | |  |  |  |
| >35 | 1.50 | 0.09 | 6.76 | 0.69 |
| <30 | 5.30 | 3.98 | 7.16 | <.001 |
| Red Blood Cell Count (m/ul) |  |  |  |  |
| >5.9 | 1.21 | 0.60 | 2.18 | 0.56 |
| <4.2 | 5.07 | 3.68 | 7.13 | <.001 |
| White Blood Cell Count (K/UL) |  |  |  |  |
| >11 | 3.38 | 2.58 | 4.48 | <.001 |
| <4.5 | 1.97 | 1.28 | 2.91 | <.001 |
| Basophils, BA# (K/UL) |  |  |  |  |
| >0.22 | 7.17 | 2.52 | 15.95 | <.001 |
| Blood Urea Nitrogen (mg/dL) |  |  |  |  |
| >20 | 5.31 | 4.04 | 7.03 | <.001 |
| <8 | 0.41 | 0.15 | 0.91 | 0.05 |
| Calcium (mg/dL) |  |  |  |  |
| >10.5 | 1.05 | 0.37 | 2.30 | 0.91 |
| <9 | 4.01 | 3.01 | 5.43 | <.001 |
| Arterial pH |  |  |  |  |
| >7.44 | 1.12 | 0.75 | 1.66 | 0.57 |
| <7.38 | 1.73 | 1.20 | 2.53 | <.001 |
| Chloride (mEq/L) |  |  |  |  |
| >106 | 4.99 | 3.70 | 6.64 | <.001 |
| <98 | 1.50 | 1.12 | 1.99 | 0.01 |
| Hemoglobin (g/dl) |  |  |  |  |
| >17 (male); >16 (female) | 1.20 | 0.51 | 2.39 | 0.63 |
| <14 (male); <12 (female) | 4.89 | 3.54 | 6.93 | <.001 |
| Lymphocytes, LY# (K/UL) |  |  |  |  |
| >4.5 | 2.29 | 1.03 | 4.37 | 0.02 |
| <0.77 | 3.73 | 2.84 | 4.93 | <.001 |
| pCO2 |  |  |  |  |
| >45 | 1.19 | 0.82 | 1.75 | 0.37 |
| <35 | 1.68 | 1.14 | 2.51 | 0.01 |
| Mean Corpuscular Volume, MCV (FL) |  |  |  |  |
| >100 | 2.55 | 1.93 | 3.34 | <.001 |
| <80 | 0.82 | 0.53 | 1.21 | 0.34 |
| Monocyte, MO# (K/UL) |  |  |  |  |
| >1.3 | 1.73 | 1.30 | 2.27 | <.001 |
| Neutrophils, NE# (K/UL) |  |  |  |  |
| >8.5 | 3.36 | 2.54 | 4.49 | <.001 |
| <2.6 | 2.18 | 1.07 | 3.93 | 0.02 |
| Anion Gap (mEq/L) |  |  |  |  |
| >11 | 3.62 | 2.63 | 4.89 | <.001 |
| <3 | 1.91 | 1.08 | 3.12 | 0.02 |
| Platelets (K/UL) |  |  |  |  |
| >400 | 2.11 | 1.34 | 3.17 | <.001 |
| <150 | 2.60 | 1.97 | 3.42 | <.001 |
| Red cell distribution width, RDW (%) |  |  |  |  |
| >14.5 | 3.22 | 2.47 | 4.19 | <.001 |
| <11.5 | 1.89 | 0.11 | 8.54 | 0.53 |
| Albumin (g/dL) |  |  |  |  |
| >5.5 | 0.82 | 0.05 | 3.71 | 0.85 |
| <3.5 | 8.78 | 6.41 | 12.21 | <.001 |
| B-type Natriuretic Peptide (pg/mL) |  |  |  |  |
| >100 | 3.05 | 1.37 | 8.65 | 0.02 |
| Thyroid Stimulating Hormone (μIU/mL) |  |  |  |  |
| >5.0 | 1.59 | 0.78 | 3.00 | 0.17 |
| <0.5 | 0.75 | 0.18 | 2.07 | 0.64 |
