# Supplementary material for: A Real-Time Early Warning System for Monitoring Inpatient Mortality Risk: Prospective Study Using Electronic Medical Record Data
Source: J Med Internet Res. 2019 Jul 5;21(7):e13719. doi: 10.2196/13719 (PMC6640073; doi:10.2196/13719)
Supplement: Multimedia Appendix 8 [file jmir_v21i7e13719_app8.docx]

Appendix 8: Odds ratios of the impactful chronic-based predictors before and after the propensity score matching analysis.

|  | Before propensity score matching | | | | After propensity score matching | | | |
| --- | --- | --- | --- | --- | --- | --- | --- | --- |
|  | OR | CI1 | CI2 | P value | OR | CI1 | CI2 | P value |
| Cardiovascular diseases | 2.74 | 2.07 | 3.68 | <.001 | 3.09 | 2.33 | 4.15 | <.001 |
| Congestive heart failure | 2.48 | 1.85 | 3.29 | <.001 | 1.51 | 1.02 | 2.25 | 0.04 |
| Myocardial infarction | 1.96 | 1.51 | 2.53 | <.001 | 1.01 | 0.76 | 1.35 | 0.94 |
| Cancer | 2.56 | 1.66 | 3.78 | <.001 | 1.62 | 0.88 | 3.06 | 0.13 |
| Renal disease | 2.16 | 1.58 | 2.90 | <.001 | 1.64 | 1.07 | 2.56 | 0.02 |
| Lung disease | 1.46 | 1.09 | 1.93 | 0.01 | 1.17 | 0.81 | 1.68 | 0.41 |
| Acute cerebrovascular disease | 1.52 | 1.05 | 2.13 | 0.02 | 0.97 | 0.62 | 1.53 | 0.91 |
