# Supplementary material for: A Real-Time Early Warning System for Monitoring Inpatient Mortality Risk: Prospective Study Using Electronic Medical Record Data
Source: J Med Internet Res. 2019 Jul 5;21(7):e13719. doi: 10.2196/13719 (PMC6640073; doi:10.2196/13719)

Appendix 9: The observed mortality rates in distinct patient subgroups, stratified by the *low-risk* (blue), *intermediate-risk* (yellow), and *high-risk* (red) categories identified by the early warning system on the prospective cohort.


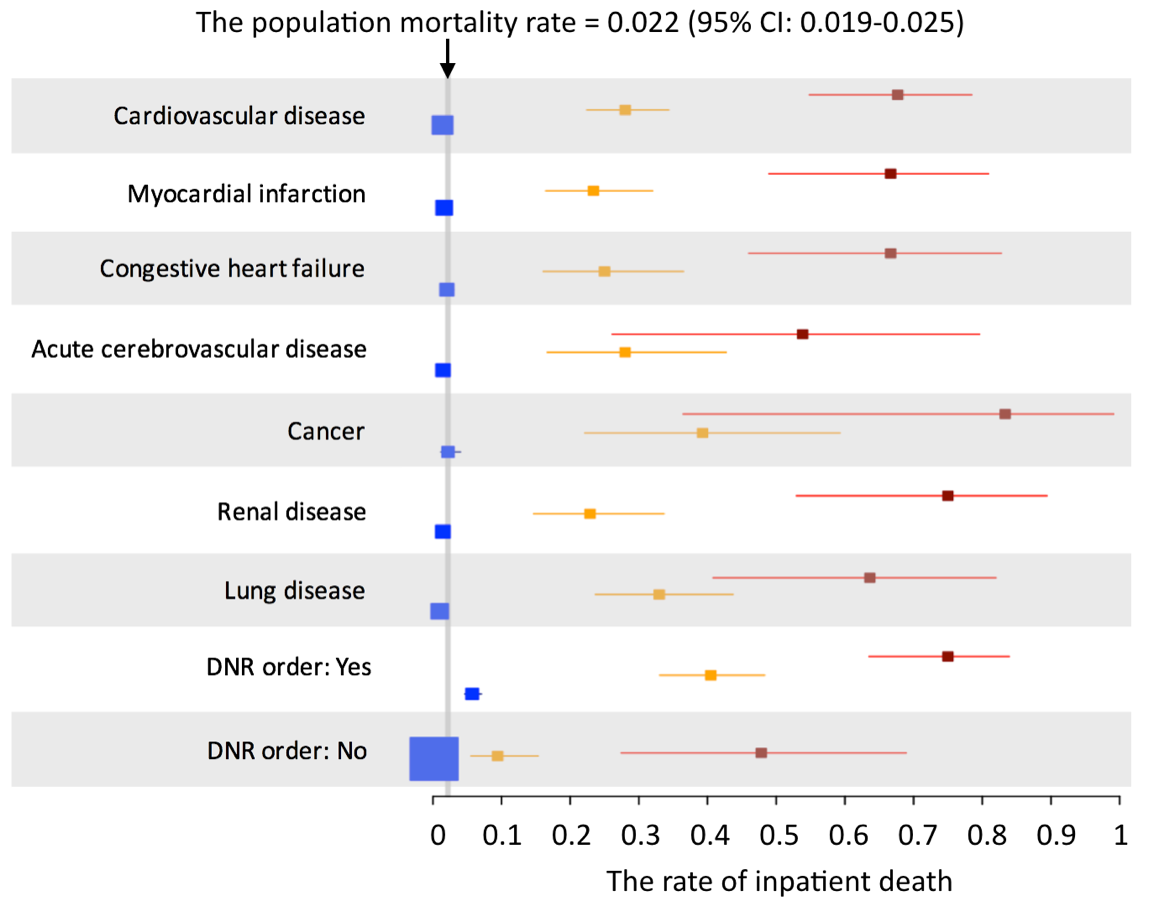

Supplement: Multimedia Appendix 9 [file jmir_v21i7e13719_app9.docx]
