# Supplementary material for: A Real-Time Early Warning System for Monitoring Inpatient Mortality Risk: Prospective Study Using Electronic Medical Record Data
Source: J Med Internet Res. 2019 Jul 5;21(7):e13719. doi: 10.2196/13719 (PMC6640073; doi:10.2196/13719)
Supplement: Multimedia Appendix 10 [file jmir_v21i7e13719_app10.docx]

Appendix 10: The inpatient mortality rate of do-not-resuscitate (DNR)-order (orange) and non–DNR-order (blue) populations in the 3 risk categories of the prospective cohort.


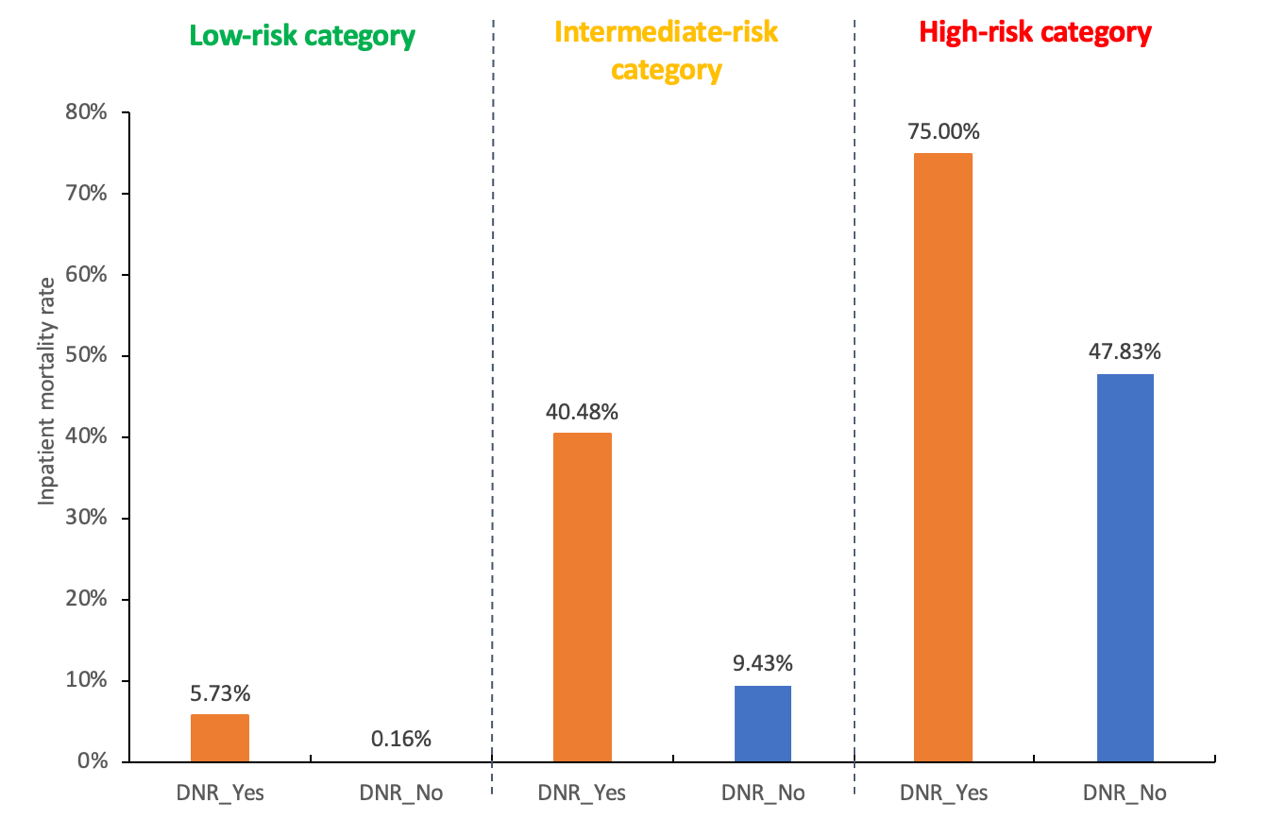


DNR: do-not-resuscitate
