# Supplementary material for: A Real-Time Early Warning System for Monitoring Inpatient Mortality Risk: Prospective Study Using Electronic Medical Record Data
Source: J Med Internet Res. 2019 Jul 5;21(7):e13719. doi: 10.2196/13719 (PMC6640073; doi:10.2196/13719)
Supplement: Multimedia Appendix 11 [file jmir_v21i7e13719_app11.docx]

Appendix 11: Age-stratified mortality across the identified risk categories.

(a) Age-stratified mortality across the identified risk categories (high-, intermediate- and low-)


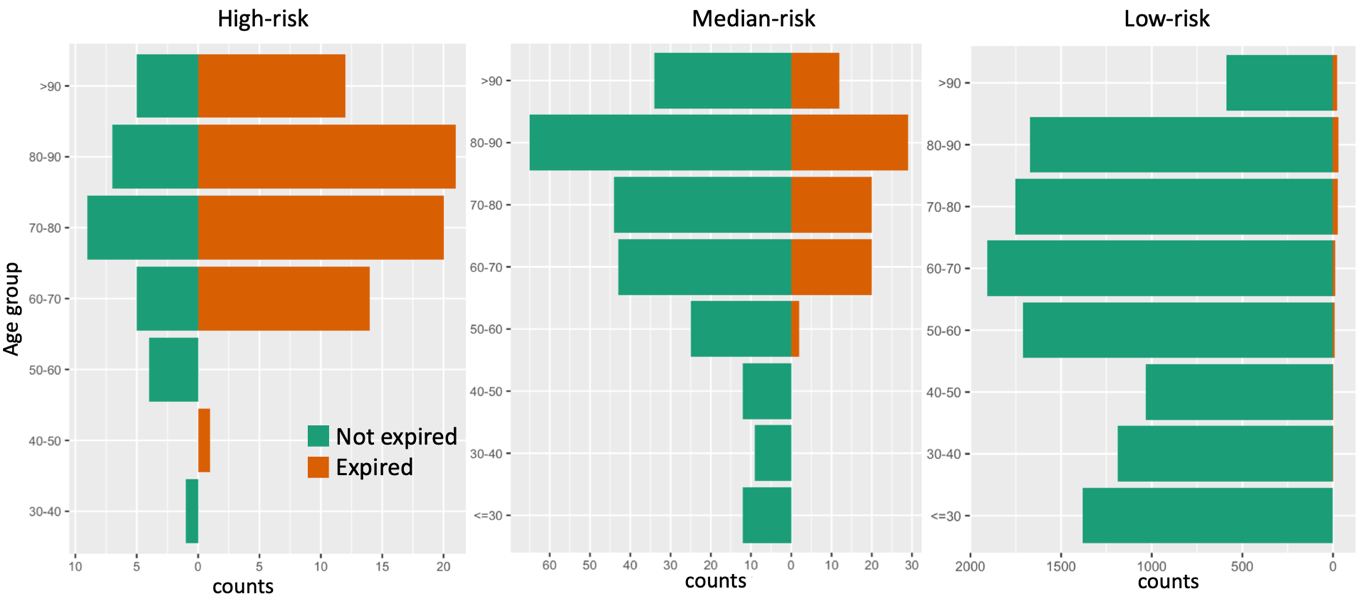


(b) The age-stratified distribution of patients with different outcomes (expired or not) across our identified category, and the numbers in parentheses are patients with fracture diagnoses.

|  | High-risk | | Median-risk | | Low-risk | |
| --- | --- | --- | --- | --- | --- | --- |
|  | Not expired | Expired | Not expired | Expired | Not expired | Expired |
| <=30 | 0 | 0 | 12 | 0 | 1,381(51) | 0 |
| 30-40 | 1 | 0 | 9(1) | 0 | 1,188(28) | 2(1) |
| 40-50 | 0 | 1 | 12 | 0 | 1,032(34) | 2(1) |
| 50-60 | 4 | 0 | 25(1) | 2(1) | 1,710(69) | 9(1) |
| 60-70 | 5 | 14 | 43(2) | 20(1) | 1,907(81) | 14(1) |
| 70-80 | 9(2) | 20(2) | 44(4) | 20(1) | 1,753(110) | 25(1) |
| 80-90 | 7(1) | 21 | 65(5) | 29(2) | 1,673(115) | 29(2) |
| >90 | 5(3) | 12 | 34(6) | 12 | 588(70) | 23(2) |
| Total | 31(8) | 68(2) | 244 | 83 | 11,232 | 104 |

(c) ICD-10 codes for fracture

|  | ICD10 code | Description |
| --- | --- | --- |
| Fractures | S02* | Fracture of skull and facial bones |
|  | S12* | Fracture of cervical vertebra and other parts of neck |
|  | S22* | Fracture of rib(s), sternum and thoracic spine |
|  | S32* | Fracture of lumbar spine and pelvis |
|  | S42* | Fracture of shoulder and upper arm |
|  | S52* | Fracture of forearm |
|  | S62* | Fracture at wrist and hand level |
|  | S72* | Fracture of femur |
|  | S82* | Fracture of lower leg, including ankle |
|  | S92* | Fracture of foot and toe, except ankle |
